# Preoperative Factors on Loss of Range of Motion after Posterior Cervical Foraminotomy

**DOI:** 10.3390/medicina60091496

**Published:** 2024-09-13

**Authors:** Dong-Ho Lee, Hyung Rae Lee, Sang Yun Seok, Ji Uk Choi, Jae Min Park, Jae-Hyuk Yang

**Affiliations:** 1 Department of Orthopedic Surgery, Asan Medical Center, University of Ulsan College of Medicine, Seoul 05505, Republic of Korea; osdlee@gmail.com (D.-H.L.); fairytales2@naver.com (J.U.C.); 2 Department of Orthopedic Surgery, Korea University Anam Hospital, Seoul 02841, Republic of Korea; kuspine@korea.ac.kr; 3 Department of Orthopedic Surgery, Daejeon Eulji Medical Center, Daejeon 34824, Republic of Korea; oper251@hanmail.net; 4 College of Medicine, Korea University, Seoul 02841, Republic of Korea; kikara02@korea.ac.kr

**Keywords:** posterior cervical foraminotomy (PCF), range of motion (ROM), bony bridge formation, neck pain VAS (visual analog scale)

## Abstract

*Background and Objectives:* Posterior cervical foraminotomy (PCF) aims to resolve cervical radiculopathy while preserving range of motion (ROM). However, its effectiveness in maintaining ROM is uncertain. This study investigates the changes in ROM after PCF and identifies preoperative factors that influence ROM reduction post surgery. *Materials and Methods:* This retrospective cohort study included patients treated at our hospital from August 2016 to September 2021. Clinical outcomes were assessed using the visual analog scale (VAS) for neck and arm pain and the neck disability index (NDI). Radiological outcomes included the segmental angle (SA), cervical angle (CA), C2–C7 SVA, Pfirrmann grade, extent of facetectomy, foraminal stenosis, and ROM. Patients were categorized into two groups based on segmental ROM changes: decreased (Group D) and maintained (Group M). Radiological and clinical outcomes were compared between the groups. Univariate and multivariate regression analyses were performed to identify risk factors for ROM loss after PCF. *Results:* 76 patients were included: 34 in Group D and 42 in Group M, with no demographic differences. Preoperatively, Group D had significantly larger flexion segmental and cervical angles than Group M (segmental, *p* < 0.001; cervical, *p* = 0.001). Group D also had a higher Pfirrmann grade (*p* = 0.014) and more bony bridge formations (*p* = 0.004). While no significant differences were observed in arm pain VAS and NDI scores, Group D exhibited worse neck pain VAS at the last follow-up (*p* = 0.03). Univariate linear regression indicated that preoperative segmental ROM (*p* < 0.001, B = 0.82) and bony bridge formation (*p* = 0.046, B = 5.33) were significant predictors of ROM loss post PCF. *Conclusions:* Patients with higher preoperative flexion angles and Pfirrmann grades at the operative level are at an increased risk for ROM loss and neck pain and often exhibit bony bridge formation. Accounting for these factors can improve surgical planning and patient outcomes.

## 1. Introduction

Posterior cervical foraminotomy (PCF) is widely recognized as an effective surgical technique to resolve cervical radiculopathy while preserving range of motion (ROM) [1,2,3,4,5]. Numerous studies have compared the outcomes of PCF and ACDF, highlighting the benefits of each approach in different clinical settings [1,2]. Compared to anterior cervical discectomy and fusion (ACDF), PCF also avoids several troublesome postoperative complications associated with ACDF, including pseudarthrosis, adjacent segmental disease, the loss of motion segments, and postoperative dysphagia [2,6,7].

However, both clinical observations and literature reviews raise doubts about the true efficacy of PCF in preserving ROM [1]. Various studies indicate that the healing process following PCF, which involves muscle stripping and facet violation, can impact alignment [8]. Clinically, there is an impression that after multi-level PCF, patients may experience a reduction in ROM, along with recurrent neck or arm pain [6,7,9,10]. This reduction in ROM can affect the patient’s quality of life (QOL) and must be considered alongside associated clinical issues. However, there is a lack of conclusive research on how ROM loss clinically impacts patient outcomes. Therefore, it is important to investigate how much ROM loss occurs after multi-level PCF, the differences in outcomes between patients with decreased and maintained ROM, and the risk factors associated with ROM reduction.

In this study, we aimed to investigate the extent and frequency of ROM reduction following PCF. Additionally, we sought to understand the clinical outcomes associated with decreased ROM and identify patient characteristics that increase the likelihood of ROM reduction post PCF.

## 2. Materials and Methods

### 2.1. Patients

The Institutional Review Board of our hospital approved this retrospective study (IRB: 2023-0592 and approval date: 18 October 2023). Informed consent was waived by the IRB due to the retrospective nature of the study. This study adhered to the Strengthening the Reporting of Observational Studies in Epidemiology (STROBE) guidelines for cohort studies. A total of 165 consecutive patients who were treated at our hospital between August 2016 and September 2021 were screened for eligibility. We excluded patients with incomplete study data or those with myelopathy, neoplasm, congenital deformity, fracture, or a history of previous cervical spinal surgery. Patients with ankylosing spondylitis (AS), ossification of the posterior longitudinal ligament (OPLL), and diffuse idiopathic skeletal hyperostosis (DISH) were also excluded as these conditions may prevent range of motion after surgery. Posterior ligament structures in the center and facet joints on the contralateral side were intact before surgery.

We included 76 patients who underwent PCF at our hospital for unilateral radiculopathy due to degenerative cervical disease and reviewed their medical records. All patients underwent a minimum of 2 years of follow-up with clinical and radiological evaluation. The demographic and radiological data were collected from the electronic medical records and the picture-archiving communication system.

To identify the predictors for postoperative ROM changes, the patients were divided into two groups based on postoperative segmental ROM changes. The decreased group (D) consisted of patients with a segmental ROM loss of more than 2 degrees after PCF, while the maintained group (M) included those whose segmental ROM decreased by 2 degrees or less, remained unchanged, or increased after PCF [6,11].

### 2.2. Surgical Procedures

PCF was performed by the senior author (DHL). All patients underwent PCF in the prone position under general anesthesia. The Mayfield head holder was applied to the head with slight cervical spine flexion. A fluoroscopic X-ray was performed to localize the exact surgical level. An incision slightly off the midline was made towards the side where radiculopathy was present, and the midline posterior ligament complex was preserved. Subperiosteal dissection was performed to minimize muscle invasion. After exposing the lamina and facet through muscular dissection, laminotomy and foraminotomy with partial facetectomy were carried out at the identified level. Special attention was given to preserving at least 50% of the lateral aspect of the facet capsule during exposure [5,12,13]. A high-speed burr was used to remove the inferior articular facet of the cephalad lamina and the superior articular facet of the caudad lamina. The exiting nerve root was then located and decompressed. If necessary, extruded or ruptured disc material was removed using pituitary forceps. The adequate decompression of the neural foramen was confirmed using a Freer elevator. The wound was closed appropriately with a closed suction drain, typically removed the following day. A soft collar was worn for the first two weeks after surgery, and patients were advised to perform gentle cervical range-of-motion exercises beginning two weeks postoperatively. These exercises included neck flexion, extension, rotation, and side bending and were continued for up to eight weeks following surgery.

### 2.3. Clinical Measures

The reviews of medical records and telephone interviews were used to evaluate the clinical outcomes. Every patient completed questionnaires to determine their neck disability index (NDI, out of 50) and visual analog scale scores for neck pain (NP, out of 10) and arm pain (AP, out of 10). These outcomes were assessed preoperatively and at 6 and 24 months during follow-up.

### 2.4. Radiological Measures

Every preoperative plain radiograph measurement was performed with the patients in neutral, flexion, and extension neck postures. The patients were instructed to stand with a horizontal gaze for the neutral position and to flex and extend their necks as much as they could tolerate for the flexion and extension lateral postures [14,15]. The same protocol was used for radiographic follow-up at 1, 6, 12, and 24 months. The assessed radiologic parameters included the segmental Cobb angle at the operative level (SA), C2–C7 Cobb angle (CA), C2–C7 sagittal vertical axis (SVA), range of motion from C2–C7 (CROM), range of motion at the operative level (SROM), segmental Cobb angle during flexion (SAF), segmental Cobb angle during extension (SAE), C2–C7 Cobb angle during flexion (CAF), and C2–C7 Cobb angle during extension (CAE). Angles were considered to represent lordosis when negative and kyphosis when positive.

**Figure 1:**
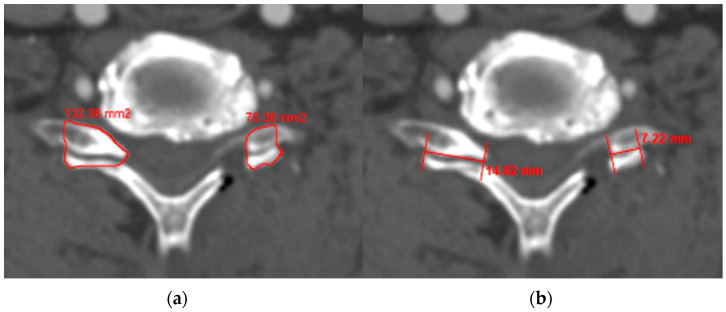
The amount of facetectomy was measured in two ways: the area (**a**) and length (**b**) of the contralateral and remnant facet.

Cervical disc degeneration was evaluated according to the Pfirrmann classification, based on the analyses of preoperative cervical MR images. This classification categorizes disc degeneration on T2-weighted images into five grades [16,17]. Foraminal stenosis at the operative level was measured using preoperative T2-weighted oblique sagittal MR images and classified into four grades [18]. In postoperative CT, we measured the area and length of the contralateral facet (a) and of the remnant facet (b) (Figure 1). The percentage of facetectomy was calculated using the following formula: Facetectomy (%) = (A − B) × 100/A [1]. The degree of joint resection was measured in the axial plane, which was reconstructed parallel to the disc level according to our standard CT protocol.

These measurements were conducted by two examiners (DWC and HRL) who were unaware of the patients’ information and not involved in their treatment. The reliability of these radiographic measurements was defined by the intraclass correlation coefficient (ICC). The ICC for interobserver reliability was 0.855, 0.88, 0.78, and 0.891 for the measurement of C2–C7 SVA, flexion cervical angle, extension cervical angle, and Pfirrmann grade.

### 2.5. Statistical Analysis

We compared radiological and clinical outcomes between Groups D (ROM decreased) and M (ROM maintained) using the independent *t*-test and chi-squared test. Time-dependent data were analyzed using repeated-measures (RM) ANOVA, followed by post hoc comparisons between the two groups. Bonferroni adjustments were applied to *p*-values to correct for multiple testing, including all pairwise comparisons within each specific model. Post hoc comparisons between the main effects of all pairs of time points were conducted. To identify risk factors for the loss of ROM after PCF, univariate and multivariate linear regression analyses were used. All statistical analyses were performed using IBM SPSS Statistics version 21.0 for Windows (IBM Corp., North Castle, NY, USA), and a *p*-value < 0.05 was considered statistically significant.

## 3. Results

### 3.1. Clinical Outcomes

**Figure 2:**
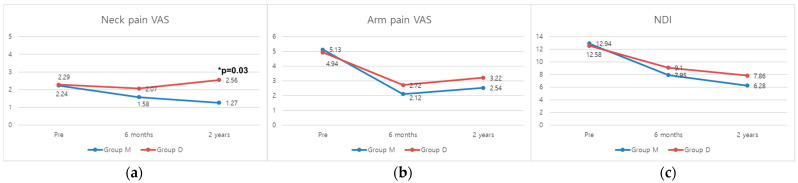
Comparisons of the mean (**a**) neck pain VAS, (**b**) arm pain VAS, and (**c**) NDI over time between Groups D and M. VAS, visual analog scale; NDI, neck disability index. * *p*-value < 0.05.

This study involved a total of 76 patients (male, *n* = 56; female, *n* = 20), with 34 patients in Group D and 42 patients in Group M. There were no significant differences between Group D and Group M in baseline characteristics, including age, sex ratio, the number of levels, bone mineral density (BMD), diabetes mellitus (DM), smoking, coronary artery disease (CAD), hospital stay, and operation time (Table 1). Group D and Group M did not show significant differences in arm pain VAS and NDI measured in all time points. However, neck pain VAS was significantly higher in Group D compared to Group M at the final follow-up (*p* = 0.03) (Figure 2).

### 3.2. Radiological Assessment

**Figure 3:**
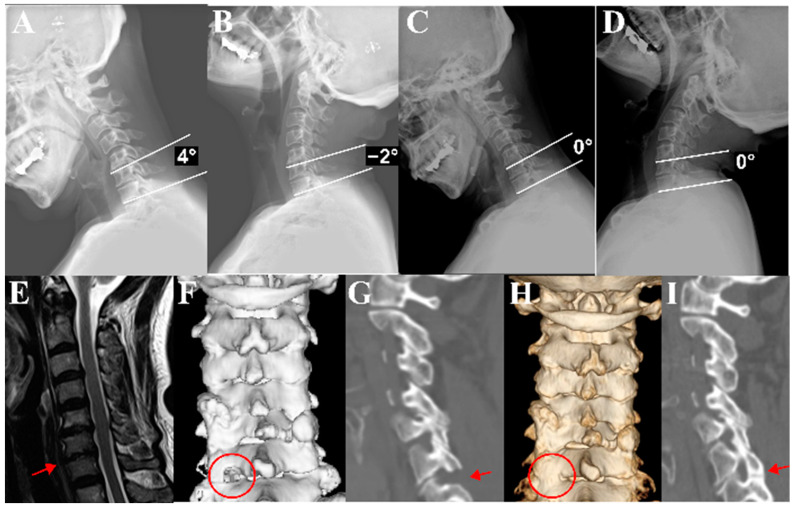
Representative patient from Group D. (**A**,**B**) Preoperative flexion cervical lateral radiograph showing a 4-degree kyphotic angle at C6–C7 and an extension lateral radiograph showing a 2-degree lordotic angle. Therefore, the preoperative segmental ROM is 6 degrees. (**C**,**D**) Postoperative flexion cervical lateral radiograph showing 0 degrees at C6–C7 and an extension lateral radiograph showing 0 degrees. Therefore, the postoperative segmental ROM is 0 degrees, resulting in a segmental ROM loss of 6 degrees. (**E**) The MRI of this patient shows Pfirrmann grade IV at C6–C7 (red arrow). (**F**,**G**) The patient’s immediate postoperative 3D CT and sagittal cut reveal the keyhole foraminotomy at C6–C7. Red circle in (**F**) and red arrow in (**G**). (**H**,**I**) The 2-year postoperative follow-up CT and 3D reconstruction show bony bridge formation. Red circle in (**H**) and red arrow in (**I**).

Preoperatively, there were no significant differences between Group D and M in segmental angle, cervical angle, and C2–C7 SVA. During flexion, Group D had significantly larger segmental and cervical angles preoperatively compared to Group M (segmental, *p* < 0.001; cervical, *p* = 0.001). However, at the last follow-up, there were no significant differences between the two groups in the flexion segmental angle and flexion cervical angle. Conversely, postoperatively at 6 months and 24 months, Group D showed a significantly smaller extension segmental angle and extension cervical angles compared to Group M (segmental at 6 M, *p* = 0.007; cervical at 6 M, *p* = 0.002; all at 2 Y, *p* < 0.001) (Table 2). This indicates that the larger preoperative flexion segmental angle decreased and the postoperative extension angle was reduced, confirming that Group D experienced a greater reduction in ROM compared to Group M. Figure 3 shows a representative patient from Group D, illustrating an example of decreased ROM after surgery. Consequently, the segmental ROM of Group D was significantly less than that of Group M at the last follow-up (*p* < 0.001). Also, Group D had larger preoperative segmental and cervical ROM than Group M (segmental, *p* < 0.001; cervical, *p* = 0.049). The changes in segmental ROM and cervical ROM from preoperative to 6 months postoperative were also significantly different between Group D and Group M (segmental, *p* < 0.001; cervical, *p* = 0.002) (Table 3). Group D had a significantly higher Pfirrmann grade (4.0 ± 0.5 vs. 3.6 ± 0.5; *p* = 0.014) and a significantly greater ratio of patients with bone bridge formation compared to Group M (61.8% vs. 26.2%; *p* = 0.004) (Table 4).

### 3.3. Factors Related to the ROM Loss after PCF

**Figure 4:**
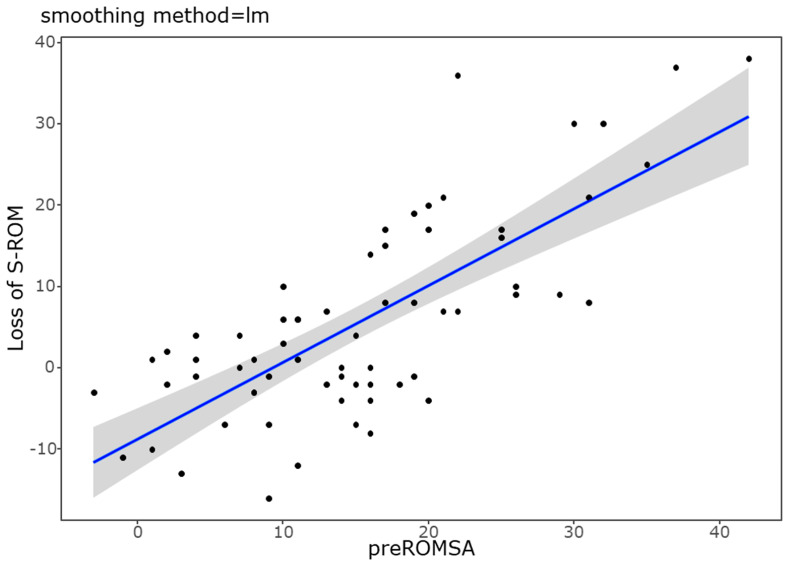
Scatter plot showing a positive correlation between increased preoperative segmental ROM and postoperative segmental ROM loss. The beta value is 0.82, and the *p*-value is <0.001.

To evaluate the risk factors for ROM reduction after surgery, we conducted univariate and multivariate linear regression analyses. In the univariate analysis, bony bridge formation (*p* = 0.046) and preoperative segmental ROM (*p* < 0.001) had a significant relationship with the loss of ROM. Multivariate linear regression with bony bridge and preoperative segmental ROM revealed that preoperative segmental ROM (*p* < 0.001, *B* = 0.8) was significantly associated with the loss of ROM (Table 5). Additionally, the positive correlation between preoperative segmental ROM and ROM loss after PCF is illustrated in the scatter plot shown in Figure 4.

## 4. Discussion

The present study aimed to evaluate the impact of PCF on changes in ROM and to identify clinical and radiological factors associated with postoperative ROM changes. Our findings provide important insights into the effects of PCF and highlight predictors for ROM loss after surgery.

Our results indicate that ROM loss is not uncommon following PCF. When comparing the group with more than 2 degrees of segmental ROM loss (Group D, *n* = 34, approximately 44.7% of the total 76 patients) to the group with less than or equal to 2 degrees of ROM loss (Group M), significant differences were observed in the preoperative flexion segmental angle and the flexion cervical angle (Table 2). Furthermore, the cervical ROM in Group D decreased from an average of 38.7 degrees preoperatively to 27.4 degrees postoperatively. Specifically, Group D exhibited greater degrees of flexion in both segmental and cervical angles preoperatively. Additionally, the Pfirrmann grade, indicative of degenerative changes, was significantly higher in Group D. These findings suggest that patients in Group D had more advanced degenerative changes and a greater degree of preoperative flexion amount, resulting in a higher preoperative ROM than Group M.

The implications of these findings are noteworthy. The greater preoperative flexion observed in Group D likely indicates a more unstable segment with progressive degenerative changes, as evidenced by the higher Pfirrmann grades in this group [19,20,21,22]. Although there was no statistically significant difference in disc height between the groups, Group D had a lower disc height, further supporting the presence of advanced degenerative changes. This instability and degeneration may predispose these patients to ROM loss after PCF. Specifically, the advanced degenerative changes, which lead to higher Pfirrmann grades, correlate with increased instability in the segment. This increased instability, combined with a larger preoperative segmental ROM, makes these segments more susceptible to significant ROM loss post surgery [21,23,24]. Moreover, our analysis indicates that an increased preoperative flexion cervical angle and advanced degenerative changes, as evidenced by higher Pfirrmann grades, are significant risk factors for ROM loss after PCF [20,25,26]. This suggests that patients with these characteristics are at a higher risk of developing postoperative arthritic changes, including bony bridges, which can diminish ROM and contribute to persistent neck pain [11,27]. Therefore, the degree of degeneration and instability in the cervical spine is a critical factor influencing postoperative ROM reduction.

An additional significant finding is the higher incidence of bony bridge formation in Group D compared to Group M (61.8% vs. 26.2%, *p* = 0.004). This supports the notion that in patients with more unstable and degenerative joints, foraminotomy may promote arthritic changes leading to bony bridge formation [5,24,28]. Such formations could contribute to the observed ROM reduction and potentially exacerbate neck pain [29,30,31]. Specifically, the formation of bony bridges resulted in a decrease in both flexion and extension angles at the affected segment, as confirmed by Table 2. Although these bony bridges did not significantly contribute to restenosis and the recurrence of arm pain (Figure 2), the increase in neck pain suggests that, similar to ACDF, PCF does not completely preserve the segmental ROM [19].

The relationship between ROM loss and neck pain is significant. Previous studies have shown that reduced cervical ROM after surgery can lead to increased neck pain and decreased quality of life (QOL) for patients. For example, a study by Meisingset et al. demonstrated that patients with greater reductions in cervical ROM experienced higher levels of neck pain [32]. Our findings align with these results as the postoperative neck pain VAS scores were significantly higher in Group D compared to Group M. This suggests that the reduction in ROM after PCF is associated with increased neck pain (Figure 2). The restricted movement resulting from ROM loss may exacerbate neck pain and may negatively impact patients’ QOL [20]. This highlights the importance of considering ROM preservation in surgical planning and patient management regarding postoperative neck pain and overall outcomes.

Importantly, our study also found that while bony bridge formation and ROM loss are linked to increased neck pain, they do not appear to worsen arm pain [19]. This suggests that while the flexibility of the cervical spine is compromised, the direct compression or irritation of nerve roots leading to arm pain is not necessarily exacerbated. This finding aligns with previous studies on restenosis, which similarly indicate that arm pain does not significantly worsen despite bony bridge formation in the foramen [19].

Despite the valuable insights gained from this study, several limitations should be acknowledged. Firstly, the retrospective nature of our study inherently limits the ability to establish causal relationships [33]. While we identified associations between preoperative characteristics and postoperative ROM changes, prospective studies are needed to confirm these findings and determine causality. Secondly, our study included a relatively small sample size from a single institution, which limits the generalizability of our findings to broader populations. Larger, multicenter studies are required to validate our results and ensure they are applicable to diverse patient populations. Thirdly, although we conducted a minimum of a 2-year follow-up, the longer-term outcomes remain unclear. Degenerative changes and their impact on ROM and clinical outcomes can evolve over time, and extended follow-up periods are necessary to fully understand the long-term implications of PCF on cervical spine biomechanics and patient quality of life [34,35]. Recognizing these limitations, future research can address these gaps and provide more robust evidence regarding the effects of PCF on cervical spine range of motion and associated clinical outcomes.

## 5. Conclusions

In conclusion, our study highlights the significance of preoperative flexion angles and degenerative changes in predicting outcomes after PCF. Patients with higher preoperative flexion angles and advanced degeneration are at a greater risk for ROM loss and neck pain due to bony bridge formation. These factors should be considered during surgical planning to improve patient outcomes. Further research is needed to develop strategies that minimize ROM loss and optimize results, especially for high-risk patients.

## Figures and Tables

**Table 1:** Baseline characteristics.

	Group D (*n* = 34)	Group M (*n* = 42)	*p*
Age (years)	63.00 ± 9.50	64.29 ± 10.67	0.710
Sex (M:F)	24:10	32:10	0.485
Level (1:2:3:4)	0:16:16:2	4:20:16:2	0.362
BMD	−0.86 ± 1.11	−0.82 ± 1.52	0.954
DM	0.78 ± 0.39	0.38 ± 0.50	0.176
Smoking	0.18 ± 0.39	0.14 ± 0.36	0.785
CAD	0.06 ± 0.24	0.14 ± 0.36	0.415
Hospital stay (days)	6.30 ± 2.80	5.80 ± 2.00	0.368
Operation time (min)	102.4 ± 34.10	95.90 ± 29.2	0.436

BMD: bone mineral density, DM: diabetes mellitus, CAD: coronary artery disease.

**Table 2:** Comparison of radiological outcomes between two groups.

	Group D (*n* = 34)	Group M (*n* = 42)	*p*
Segmental Angle (°)			
Preoperative	−0.3 ± 6.0	−2.8 ± 5.4	0.089
Postoperative 6 M	−0.5 ± 5.6	−4.1 ± 6.5	0.021 *
Postoperative 2 Y	−0.1 ± 4.6	−3.8 ± 6.1	0.012 *
Cervical Angle (°)			
Preoperative	−5.0 ± 9.0	−9.0 ± 10.1	0.098
Postoperative 6 M	−4.6 ± 8.0	−11.8 ± 11.5	0.006 *
Postoperative 2 Y	−4.7 ± 8.8	−13.0 ± 12.3	0.005 *
C2–C7 SVA (mm)			
Preoperative	21.0 ± 10.4	21.6 ± 12.5	0.816
Postoperative 6 M	21.2 ± 9.5	17.9 ± 11.7	0.211
Postoperative 2 Y	19.8 ± 11.8	19.1 ± 10.6	0.822
Flexion Segmental Angle (°)			
Preoperative	12.6 ± 6.9	3.9 ± 7.5	<0.001 *
Postoperative 6 M	9.2 ± 6.3	5.2 ± 5.5	0.010 *
Postoperative 2 Y	7.0 ± 7.5	6.2 ± 5.6	0.641
Extension Segmental Angle (°)			
Preoperative	−7.8 ± 7.5	−5.9 ± 6.8	0.287
Postoperative 6 M	−3.7 ± 6.4	−8.0 ± 5.7	0.007 *
Postoperative 2 Y	−0.8 ± 6.0	−8.5 ± 6.7	<0.001 *
Flexion Cervical Angle (°)			
Preoperative	23.6 ± 10.0	11.4 ± 16.0	0.001 *
Postoperative 6 M	17.2 ± 10.4	13.6 ± 12.8	0.216
Postoperative 2 Y	16.2 ± 12.5	14.3 ± 10.6	0.548
Extension Cervical Angle (°)			
Preoperative	−15.2 ± 13.2	−18.8 ± 14.2	0.293
Postoperative 6 M	−11.9 ± 10.6	−20.8 ± 11.0	0.002 *
Postoperative 2 Y	−10.2 ± 10.1	−22.8 ± 12.3	<0.001 *

* *p* < 0.05. A negative value indicates a lordotic angle, while a positive value indicates a kyphotic angle.

**Table 3:** ROM change by time between two groups.

	Group D (*n* = 34)	Group M (*n* = 42)	*p*
Pre-segmental ROM	20.4 ± 9.6	9.8 ± 6.3	<0.001 *
6 M segmental ROM	12.2 ± 6.6	12.8 ± 7.2	0.714
ROM change (pre-6 M)	−8.2 ± 8.6	3.0 ± 6.9	<0.001 *
2 Y segmental ROM	6.1 ± 7.3	13.7 ± 7.1	<0.001 *
ROM change (6 M-2 Y)	−6.2 ± 7.9	0.9 ± 6.3	<0.001 *
Pre-cervical ROM	38.7 ± 15.5	30.2 ± 18.9	0.049 *
6 M cervical ROM	20.8 ± 15.7	34.7 ± 17.0	0.001 *
ROM change (pre-6 M)	−18.3 ± 18.7	3.1 ± 17.8	0.002 *
2 Y cervical ROM	27.4 ± 13.9	33.2 ± 15.4	0.114
ROM change (6 M-2 Y)	7.2 ± 17.7	−1.5 ± 13.5	0.030 *

Pre: preoperative, ROM: range of motion. * *p* < 0.05.

**Table 4:** Comparison of radiologic factors between two groups.

	Group D (*n* = 34)	Group M (*n* = 42)	*p*
Disc height (mm)	3.6 ± 0.9	3.9 ± 0.8	0.161
Gliding distance (mm)	1.9 ± 3.3	1.4 ± 0.8	0.452
Pre-foraminal dimension (mm^2^)	31.5 ± 7.8	34.3 ± 8.5	0.164
Post-foraminal dimension (mm^2^)	53.3 ± 7.4	54.5 ± 8.2	0.517
Foraminal enlargement (%)	75.5 ± 34.6	63.8 ± 26.1	0.132
Foraminal stenosis grade	3.7 ± 0.4	3.6 ± 0.5	0.103
Pfirrmann grade	4.0 ± 0.5	3.6 ± 0.5	0.014 *
Facetectomy, area (%)	52.9 ± 12.9	46.9 ± 15.9	0.101
Facetectomy, width (%)	49.5 ± 10.3	46.1 ± 11.7	0.451
Bone bridge (*n*, %)	21 (61.8%)	11 (26.2%)	0.004 *

* *p* < 0.05.

**Table 5:** Univariate and multivariate linear regression analysis for ROM change after PCF.

Variable	Univariate		Multivariate		
	B (SE)	*p*	B (SE)	95% CI	*p*
				(R^2^ = 0.5755, *p* < 0.001)	
Bone bridge	5.33 (2.71)	0.046 *	1.53 (2.06)	−0.15–0.32	0.118
Facetectomy (%)	0.01 (0.11)	0.903			
Preop SA	−0.04 (0.3)	0.881			
Preop CA	0.25 (0.18)	0.184			
C2–C7 SVA	−0.24 (0.15)	0.117			
Pre-segmental ROM	0.82 (0.12)	<0.001 *	0.80 (0.12)	0.51–0.94	<0.001 *
Disc height	−0.81 (1.91)	0.675			
Gliding distance	0.22 (0.54)	0.683			
Pfirmann grade	4.38 (2.94)	0.145			
Foraminal stenosis grade	5.53 (4.03)	0.178			
Preop foraminal area	−0.31 (0.21)	0.142			
Foraminal enlargement (%)	0.02 (0.05)	0.724			
Number of levels	1.76 (2.62)	0.506			

ROM: range of motion, PCF: posterior cervical foraminotomy, SE: standard error, CI: confidence interval, Pre: preoperative, SA: segmental angle, CA: cervical angle, SVA: sagittal vertical axis. * *p* < 0.05.

## Data Availability

The raw data supporting the conclusions of this article will be made available by the authors on request.
